# Mendelian randomization supports causality between overweight status and accelerated aging

**DOI:** 10.1111/acel.13899

**Published:** 2023-06-05

**Authors:** Zong Chen, Zhiyou Chen, Xiaolei Jin

**Affiliations:** ^1^ 16th Department, Plastic Surgery Hospital Chinese Academy of Medical Sciences and Peking Union Medical College Beijing China; ^2^ 8th Department, Plastic Surgery Hospital Chinese Academy of Medical Sciences and Peking Union Medical College Beijing China

**Keywords:** facial aging, frailty index, Mendelian randomization, overweight, telomere length

## Abstract

It is reported that overweight may lead to accelerated aging. However, there is still a lack of evidence on the causal effect of overweight and aging. We collected genetic variants associated with overweight, age proxy indicators (telomere length, frailty index and facial aging), etc., from genome‐wide association studies datasets. Then we performed MR analyses to explore associations between overweight and age proxy indicators. MR analyses were primarily conducted using the inverse variance weighted method, followed by various sensitivity and validation analyses. MR analyses indicated that there were significant associations of overweight on telomere length, frailty index, and facial aging (*β* = −0.018, 95% CI = −0.033 to −0.003, *p* = 0.0162; *β* = 0.055, 95% CI = 0.030–0.079, *p* < 0.0001; *β* = 0.029, 95% CI = 0.013–0.046, *p* = 0.0005 respectively). Overweight also had a significant negative causality with longevity expectancy (90th survival percentile, *β* = −0.220, 95% CI = −0.323 to −0.118, *p* < 0.0001; 99th survival percentile, *β* = −0.389, 95% CI = −0.652 to −0.126, *p* = 0.0038). Moreover, the findings tend to favor causal links between body fat mass/body fat percentage on aging proxy indicators, but not body fat‐free mass. This study provides evidence of the causality between overweight and accelerated aging (telomere length decreased, frailty index increased, facial aging increased) and lower longevity expectancy. Accordingly, the potential significance of weight control and treatment of overweight in combating accelerated aging need to be emphasized.

## Graphical abstract

This study provides evidence of the causality between overweight and accelerated aging (telomere length decreased, frailty index increased, facial aging increased). Moreover, the findings support the association of overweight and decreased longevity expectancy (lower likelihoods to reach or exceed 90th survival percentile and 99th survival percentile). Accordingly, the potential significance of weight control and treatment of overweight in combating accelerated aging need to be emphasized.

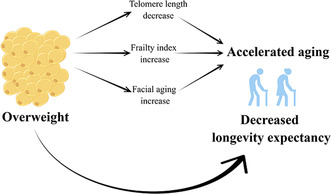

**Abbreviations**
BFFM: body fat‐free mass
BFM: body fat mass
BFP: body fat percentage
BMI: body mass index
BW: body weight
CHD: coronary heart disease
DBP: diastolic blood pressure
FA: facial aging
FI: frailty index
GIANT: Genetic Investigation of ANthropometric Traits
GSCAN: GWAS and Sequencing Consortium of Alcohol and Nicotine use
GWASes: genome‐wide association studies
HbA1c: glycosylated hemoglobin
HOMA‐IR: homeostasis model assessment of insulin resistance
IS: ischemic stroke
IV: instrumental variable
IVW‐FE: fixed effects inverse variance weighted
IVW‐MRE: multiplicative random effects inverse variance weighted
MAGIC: the Meta‐Analyses of Glucose and Insulin‐Related Traits Consortium
MR: Mendelian randomization
MR‐PRESSO: Mendelian randomization pleiotropy residual sum and outlier
OR: odd ratio
PL: parental lifespans
qPCR: quantitative polymerase chain reaction
RCTs: randomized controlled trials
SBP: systolic blood pressure
SC: serum creatinine
SNP: single nucleotide polymorphism
TL: telomere length
WHO: World Health Organization

## 1 INTRODUCTION

Aging is the accumulation of aging cells in organisms, accompanied by the decline of biological functions and a series of prominent features, including genetic and epigenetic changes (Wang et al., 2022). Among them, telomere shortening and damage is one of the microcosmic manifestations of cell aging and human aging (Rossiello et al., 2022). In addition, chronic diseases, frailty, cognitive dysfunction and facial aging (FA) are important characteristics of aging (Franco et al., 2022; Gonzales et al., 2022). Accelerated aging means that the biological age of the body exceeds the actual age, which will undoubtedly lead to increased risk of disease and death, and reduced life expectancy and quality of life (Belsky et al., 2015). Biological age proxy indicators are needed to assess accelerated aging, including telomere length (TL), epigenetic clock and frailty index (FI) (Hoogendijk et al., 2019; Jylhävä et al., 2017). Effective identification and control of factors that accelerate aging will help prevent premature death, extend healthy life expectancy and improve quality of life.

The condition of being overweight is defined as abnormal or excessive fat accumulation that may impair health. According to World Health Organization (WHO) standard, it is defined as body mass index (BMI) ≥ 25 kg/m^2^ (World Health Organization, 2000). The WHO reported the number of overweight and obesity has doubled in the past few decades (Caballero, 2019). Studies confirm that overweight increases the risk of cardiovascular disease, diabetes and cancers, and overweight and obesity has been identified as one of the most serious public health problems of the 21st century (Iyengar et al., 2016; Piché et al., 2020). Evidence suggests that overweight may accelerate aging (Santos & Sinha, 2021). But the causality between overweight and aging has not been identified.

Limited by the quality of evidence, possible potential reverse causality and residual confounding, observational studies have been almost unable to identify a causal association between overweight and aging (Hoffmann et al., 2018). In this regard, randomized controlled trials (RCTs) can be used to reveal cause and effect (Stanley, 2007). However, RCTs are costly in terms of money, time and manpower, and some interventions are not approved or are not suitable for RCTs assessment. Mendelian randomization (MR) is a popular and effective method for causal inference in recent years. It takes genetic variation (single nucleotide polymorphism, SNP) as the instrumental variable (IV) to deduce the causal association between outcome and exposure, which can effectively avoid the confounding bias of traditional epidemiological studies (Sekula et al., 2016).

We performed the present MR study with the aim of evaluating the causality between overweight and aging by analyzing the summary‐level genome‐wide association studies (GWASes) data of overweight, age proxy indicators such as TL, FI and FA, and other traits, etc.

## 2 METHODS

### 2.1 Data source

Genetic variants significantly associated with overweight were extracted from a large GWAS of Genetic Investigation of ANthropometric Traits (GIANT) consortium, which comprised 93,105 cases and 65,840 controls (Berndt et al., 2013). Among them, the definition of overweight (case) and normal weight (control) were based on a baseline measurement of BMI and used the WHO standards (World Health Organization, 2000). Therefore, the inclusion criterion for cases was BMI ≥25 kg/m^2^, while for controls was 18.5 kg/m^2^ ≤ BMI < 25 kg/m^2^.

The genetic variants associated with TL and FA were from UK Biobank with the sample sizes of 472,174 (216,187 males and 255,987 females, age 56.1 ± 7.9) and 423,999 (194,391 males and 229,601 females, age 40–69). At the UK Biobank, the mean leukocyte TL was measured in the mixed leukocyte population by using the multiplex quantitative polymerase chain reaction (qPCR) technique, which expressed the TL as the ratio of telomere repeats to single copy genes (T/S ratio) (Codd et al., 2022). The logarithmic distribution is then converted to approximate normal distribution. Then paired LTL measurements were made from DNA taken at two time‐points (mean interval: 5.5 years) in 1351 participants to enable calculation of, and correction for, regression‐dilution. The log_e_‐transformed leukocyte TL was 0.68 ± 0.02, and the study estimated that at age 40 years, people with >1‐SD shorter compared to ≥1‐SD longer leukocyte TL than the population mean had 2.5 years lower life expectancy. FA was assessed with non‐subjective perceived age based on questionnaire. The results showed that 8630 reported looking older than their biological age, 103,300 reported looking about their age, and 312,062 reported looking younger than their biological age. For this analysis, participants were coded 1 if they reported that they looked younger, 0 if they reported that they looked older, and 0.5 if they reported that they looked their age (Observations were made by third parties, both non‐participants and non‐researchers, who did not know the actual age of the participants. The researchers coded the participants' FA according to their perceived age and actual age). Using a mixed linear model analysis (which could test the relationship between genotype and phenotype while accounting for covariates (age, sex, and study participation center) and relatedness), FA can be identified as an ordered categorical variable. Then statistics on the linear scale were transformed into log odd ratio (OR) using a Taylor expansion series. OR >1 indicate greater odds of looking youthful (Jiang et al., 2019). Genetic variants significantly associated with the FI were obtained from a GWAS meta‐analysis with 164,610 (79,791 males and 84,819 females, age 64.1 ± 2.8) UK Biobank participants and 10,616 (5039 males and 5577 females, age 58.3 ± 7.9) TwinGene participants by Atkins et al. (2021). Rockwood FI based on deficit accumulation model is used as the outcome measure of frailty. A score of 0 or 1 was assigned according to the amount of compliance with the deficit (0 means no). Accordingly, the FI of each person is calculated as the number of deficits divided by the total number of 49 deficits described in the previous study (Table S1). The greater the value of the FI, the more serious the individual's frailty. The results showed that mean proportion of deficits in UK biobank and TwinGene participants were 0.129 ± 0.075 and 0.121 ± 0.080, respectively. Genetic variants associated with the longevity were obtained from two GWAS meta‐analyses that included 11,262/3484 cases surviving at or beyond the age corresponding to the 90th/99th survival percentile, respectively, and 25,483 controls. In the study, cases were individuals who lived to an age above the 90th or 99th percentile based on cohort life tables from census data from the appropriate country, sex, and birth cohort. Controls were individuals who died at or before the age at the 60th percentile or whose age at the last follow‐up visit was at or before the 60th percentile age (Deelen et al., 2019).

Other GWAS datasets obtained in our study included: homeostasis model assessment of insulin resistance (HOMA‐IR) with 37,037 subjects from Dupuis et al. (2010); body weight (BW), body fat mass (BFM), body fat percentage (BFP) and body fat‐free mass (BFFM) with 336,227 subjects, 330,762 subjects, 331,117 subjects and 331,291 subjects from Neale Lab consortium; parental lifespans (PL) with 1,012,240 subjects from Timmers et al. (2019); waist circumference, hip circumference, waist‐to‐hip ratio and BMI with 232,102 subjects, 213,038 subjects, 212,244 subjects and 339,224 subjects from GIANT consortium; cigarettes per day and alcoholic drinks per week with 337,334 subjects and 335,394 subjects from GWAS and Sequencing Consortium of Alcohol and Nicotine use (GSCAN); coronary heart disease (CHD) with 30,482 subjects from Coronary Artery Disease (C4D) Genetics Consortium; glycosylated hemoglobin (HbA1c) with 46,368 subjects from the Meta‐Analyses of Glucose and Insulin‐Related Traits Consortium (MAGIC); systolic blood pressure (SBP) and diastolic blood pressure (DBP) with 757,601 subjects from the International Consortium of Blood Pressure consortium; ischemic stroke (IS) with 440,328 subjects from Malik et al. (2018); serum creatinine (SC) with 133,814 subjects from CKDGen Consortium.

All the exposure and outcome datasets were of European ancestry or mainly composed of European ancestry. There was no large‐scale crossover and overlap between participants that included in GWAS of overweight and GWASes of aging proxy indicators and longevity.

The present study only used publicly available summary‐level statistics. Ethical approval is therefore not required.

### 2.2 IV selection criteria

SNPs significantly associated with exposures or outcome (*p* < 5 × 10^−8^) were selected as IVs from the GWAS datasets, respectively. Then, we pruned the candidate IVs for linkage disequilibrium (*r* ^2^ > 0.001) and discarded variants that were within 1‐Mb distance from other IVs with a stronger association. *R* ^2^, the proportion of exposure explained by IVs, can be calculated by the formula: *R* ^2^ = 2 × *β* ^2^ × EAF × (1 − EAF), where *β* was the estimated effect size of the SNPs and EAF indicated effect allele frequency. *F*‐statistic is a common index to evaluate weak instrumental bias, can be calculated by the following formula: *F* = *R* ^2^/(1 − *R* ^2^) × (*N*–*k* − 1)/*k*, where *N* was the sample size and *k* was the number of included SNPs. When the *F*‐statistic <10, we consider the genetic variation used as a weak IV, which may produce a certain bias to the results, so SNPs with *F*‐statistic <10 will be excluded.

### 2.3 Statistical analysis

The present study was conducted in the R software (version 4.2.1, The R Development Core Team, Vienna, Austria), we used base (version 4.2.1), TwoSampleMR (version 0.5.6), MRInstruments (version 0.3.2), MRPRESSO (version 1.0) MendelianRandomization (version 0.6.0), data table (version 1.14.2) and ggplot2 (version 3.3.6) R package and related functions.

For two‐sample MR analysis, we evaluated the causal links between exposures (overweight, BW, BFM, BFP, BFFM and HOMA‐IR, etc.) and outcomes (aging proxy indicators, longevity and PL, etc.) by fixed effects inverse variance weighted (IVW‐FE) method. We also used the simple median, simple mode, weighted mode, weighted median, MR Egger and MR pleiotropy residual sum and outlier (MR‐PRESSO) methods for additional analysis. Sensitivity analyses were performed to verify and adjust the validity and stability of the results, which included heterogeneity test (Cochrane's *Q* test, MR‐PRESSO global test), pleiotropy test (MR Egger intercept test, MR‐PRESSO distortion test), and leave‐one‐out test (Bowden et al., 2015; Emdin et al., 2017; Hemani et al., 2018; Verbanck et al., 2018). Once heterogeneity was identified (*p* < 0.05), the multiplicative random effects IVW (IVW‐MRE) method should be used for assessing the causal effect.

Although a series of statistical methods have been carried out in the sensitivity analyses, we used Phenoscanner V2 (http://www.phenoscanner.medschl.cam.ac.uk/) for a confounding analysis (Staley et al., 2016). We explored diseases/physical conditions that are significantly related to the including SNPs at the threshold of *p* < 1 × 10^−5^ (No clear confounding factor was found at the threshold of *p* < 5 × 10^−8^), then summarize and analyze the related information about the SNPs, GWASes and Diseases. This not only helps to identify potential confounders for adjustment in multivariate analysis, but also helps us to explore the mediation and potential mechanism of causality.

For multivariable MR analysis, pooled several factors (mainly from confounding analysis, including cigarettes per day, alcoholic drinks per week, HbA1c, SBP, DBP, CHD, IS and SC) in the analysis for adjustment. The IVW method was used for the multivariable analysis. Bonferroni correction was used for multiple comparisons, and its critical *p* value was defined in relation to the number of exposures and outcomes, following the formula: *p* = 0.05/*E*/*C* (*E* and *C* were the number of exposures and outcomes, respectively).

We used a mediation MR analysis (two‐step MR) to verify and analyze the mediators that mediated the associations between overweight and aging proxy indicators. Candidate mediators were mainly HbA1C, CHD, and IS (it should be noted that they were included in the deficits of FI). The specific method includes two steps. Step (1): to find significant SNPs from the GWAS about exposure, remove SNPs with linkage disequilibrium, and then extract the remaining SNPs from the GWAS of the mediating variable. It is necessary to ensure that the remaining SNPs are not directly related to confounding factors and mediating variable. Finally, the causal effect of exposure on mediator (assume beta1) are calculated. Step (2): Use the same method to calculate the causal effect of mediator on outcome (assume it is beta2). Assume that the causal effect of exposure on outcome is beta0. The following conditions exist: (i) If beta0, beta1, and beta2 are all significant, this indicates that there is a causal association between exposure and outcome, and this association may be partially mediated by mediating variables. beta1*beta2 can be used as the mediating effect from exposure to outcome, and its mediating proportion can also be calculated ((beta1*beta2)/beta0). (ii) If beta0 is not significant, but beta1 and beta2 are both significant, the association from exposure to outcome can be considered to be completely mediated by this mediator. (iii) If beta0 is significant, but at least one of beta1 and beta2 is not significant, there is no mediating effect mediated by this mediating variable in the causal association of exposure on outcome (Relton & Davey Smith, 2012).

## 3 RESULTS

### 3.1 Overweight and aging proxy indicators

**FIGURE 1:**
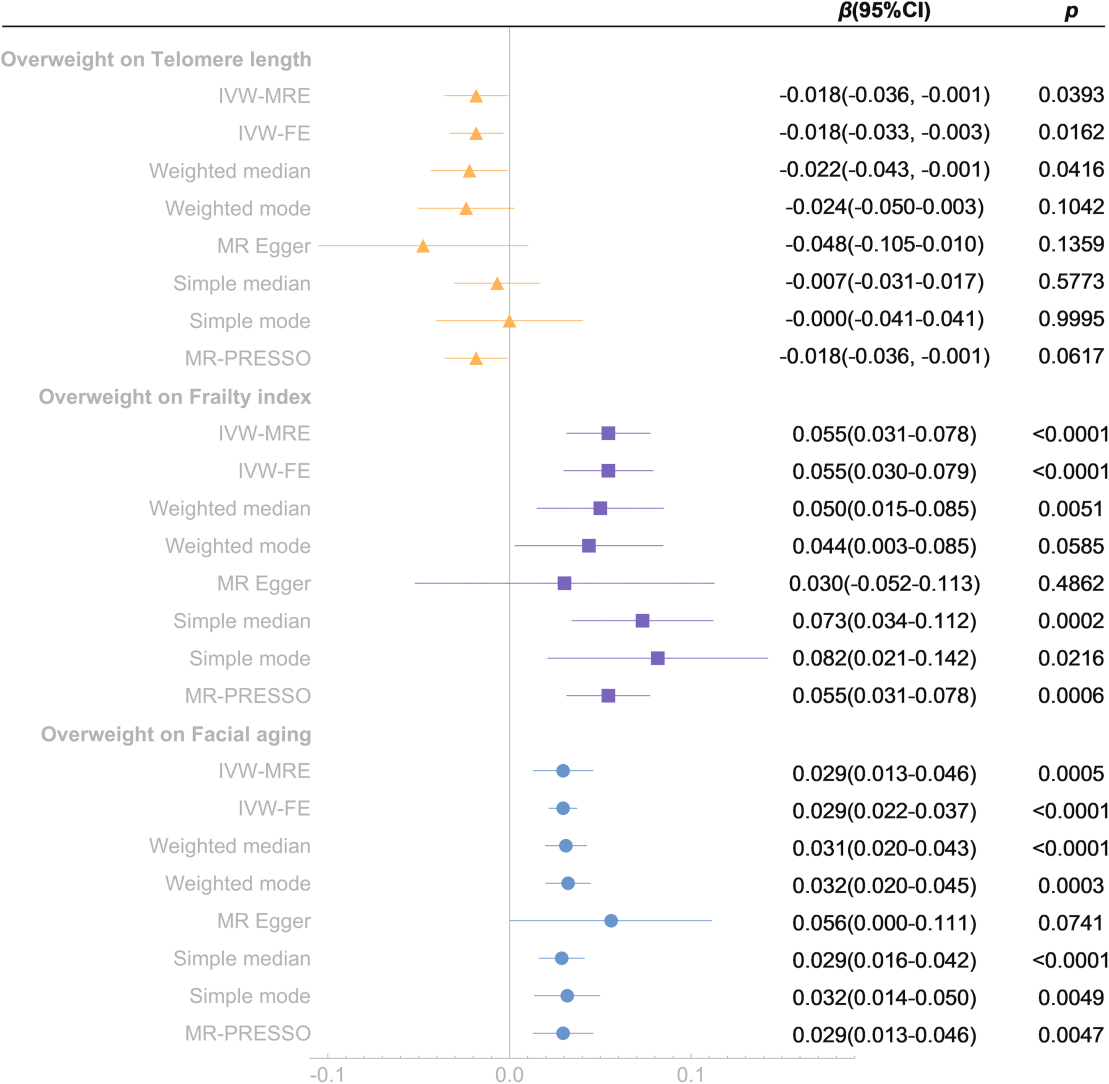
Mendelian randomization analysis of the effect of overweight on telomere length, frailty index and facial aging. IVW‐MRE, inverse variance weighted (multiplicative random effects); IVW‐FE, inverse variance weighted (fixed effects); MR‐PRESSO, Mendelian randomization pleiotropy residual sum and outlier.

Fourteen SNPs associated with overweight at genome‐wide significance were identified, and one weak IV (rs12444979, *F* = 7.99) was excluded (Table 1). The main results of MR analysis are shown in Figure 1. IVW‐FE method indicated that there were significant causal associations of genetically predictive overweight on TL, FI and FA (*β* = −0.018, 95% confidence interval [CI] = −0.033 to −0.003, *p* = 0.0162; *β* = 0.055, 95% CI = 0.030–0.079, *p* < 0.0001; *β* = 0.029, 95% CI = 0.022–0.037, *p* < 0.0001 respectively). Overweight was significantly associated with decreased telomere length, increased FI and FA (Figure 1). Sensitivity analysis showed that there was heterogeneity in the result of FA, but there was no pleiotropy (Table 2). The further IVW‐MRE method was further used and indicated causal effect of overweight on FA (*β* = 0.029, 95% CI = 0.013–0.046, *p* = 0.0005). Leave‐one‐out tests suggested that the associations between overweight and TL, FI, and FA were effective and sensitive, while the association between overweight and TL was less robust (Figure S1). We further analyzed the associations between every single SNP (associated with overweight) and TL, and the results showed that a few SNPs (rs9816226, rs10182181, rs10853932 and rs13130484) were associated with TL increase (Figure S2).

**TABLE 1:** Included SNPs that are significantly associated with overweight.

SNP	Nearby gene	Chr.	EA	OA	EAF	*β*	SE	*p*‐value	*R* ^2^	*F* statistic
rs10182181	*ADCY3*	2	G	A	0.50	0.057	0.009	2.10E‐10	0.0016	19.88
rs10853932	*KCTD15*	19	C	T	0.69	0.067	0.011	1.30E‐09	0.0019	23.47
rs12444979*	*GPRC5B*	16	T	C	0.06	−0.079	0.013	1.80E‐09	0.0007	7.99
rs12623218	*TMEM18*	2	A	T	0.88	0.110	0.012	5.80E‐22	0.0025	31.08
rs13130484	*GNPDA2*	4	T	C	0.42	0.071	0.009	3.90E‐14	0.0025	30.16
rs1421085	*FTO*	16	C	T	0.45	0.140	0.009	5.80E‐50	0.0097	119.61
rs2030323	*BDNF*	11	C	A	0.78	0.079	0.011	1.10E‐12	0.0021	25.97
rs2206277	*TFAP2B*	6	T	C	0.10	0.080	0.012	5.60E‐12	0.0011	13.59
rs2568958	*NEGR1*	1	A	G	0.65	0.062	0.009	1.10E‐11	0.0017	21.41
rs2596125	*HNF4G*	8	T	C	0.44	−0.052	0.009	5.90E‐09	0.0013	16.32
rs523288	*MC4R*	18	T	A	0.29	0.099	0.011	1.70E‐20	0.0040	49.31
rs633715	*SEC16B*	1	C	T	0.27	0.078	0.011	4.00E‐12	0.0024	29.17
rs8028313	*MAP2K5*	15	G	C	0.22	−0.065	0.011	2.00E‐09	0.0015	17.74
rs9816226	*ETV5*	3	T	A	0.85	0.070	0.012	2.00E‐09	0.0012	15.20

Abbreviations: Chr, chromosome; EA, effect allele; EAF, effect allele frequency; OA, other allele; SE, standard error; SNP, single nucleotide polymorphism.

* Weak instrumental variable.

**TABLE 2:** Sensitivity analysis of overweight on facial aging, frailty index, telomere length, 90th survival percentile, and 99th survival percentile.

Overweight on	Heterogeneity test				Pleiotropy test			
	Cochrane's *Q* test		MR‐PRESSO global test		MR‐Egger intercept test		MR‐PRESSO distortion test	
	*Q*	*p*	RSSobs	*p*	Intercept	*p*	Coefficient	*p*
Facial aging	53.4987	<0.01	60.2901	<0.01	−0.0023	0.349	−4.0054	0.815
Frailty index	10.4656	0.575	12.2154	0.630	0.0021	0.559	0	1
Telomere length	16.3291	0.177	18.9872	0.187	0.0025	0.322	0	1
90th survival percentile	6.1065	0.911	6.9130	0.918	0.0083	0.685	0	1
99th survival percentile	16.3701	0.175	18.6994	0.216	0.0310	0.420	0	1

We also conducted reverse association analyses of aging proxy indicators on overweight, which showed no reverse causality (Figure S3).

### 3.2 Overweight and longevity

**FIGURE 2:**
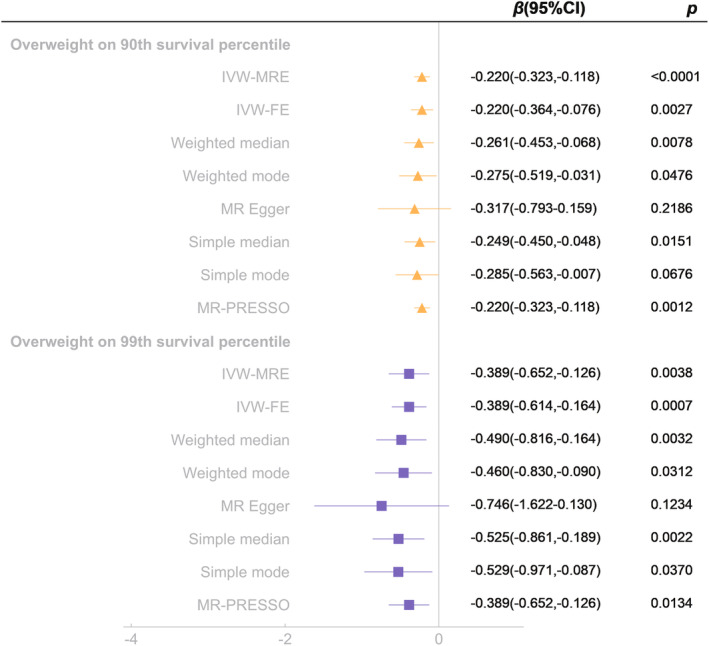
Mendelian randomization analysis of the effect of overweight on longevity (90th survival percentile and 99th survival percentile). IVW‐MRE, inverse variance weighted (multiplicative random effects); IVW‐FE, inverse variance weighted (fixed effects); MR‐PRESSO, Mendelian randomization pleiotropy residual sum and outlier.

MR analyses showed that there were significant causal associations of overweight on longevity (90th survival percentile and 99th survival percentile). Overweight was associated with decreased longevity expectancy (90th survival percentile, *β* = −0.220, 95% CI = −0.323 to −0.118, *p* < 0.0001; 99th survival percentile, *β* = −0.389, 95% CI = −0.652 to −0.126, *p* = 0.0038) (Figure 2). Sensitivity analysis showed that there was no heterogeneity and pleiotropy (Table 2). Moreover, leave‐one‐out tests indicated that the associations were effective and robust (Figure S4).

### 3.3 Overweight and PL

There was significant causal link of overweight on PL (*β* = −0.129, 95% CI = −0.173 to −0.084, *p* < 0.0001), but the reverse causality was not significant (Figure S5).

### 3.4 HOMA‐IR, overweight and aging proxy indicators

The analyses results showed a causal association between HOMA‐IR and overweight (*β* = 0.606, 95% CI =0.402–0.810, *p* < 0.0001), but there was no causality of HOMA‐IR on aging proxy indicators (Figure S6).

### 3.5 BW, BFM, BFP, BFFM and aging proxy indicators

**FIGURE 3:**
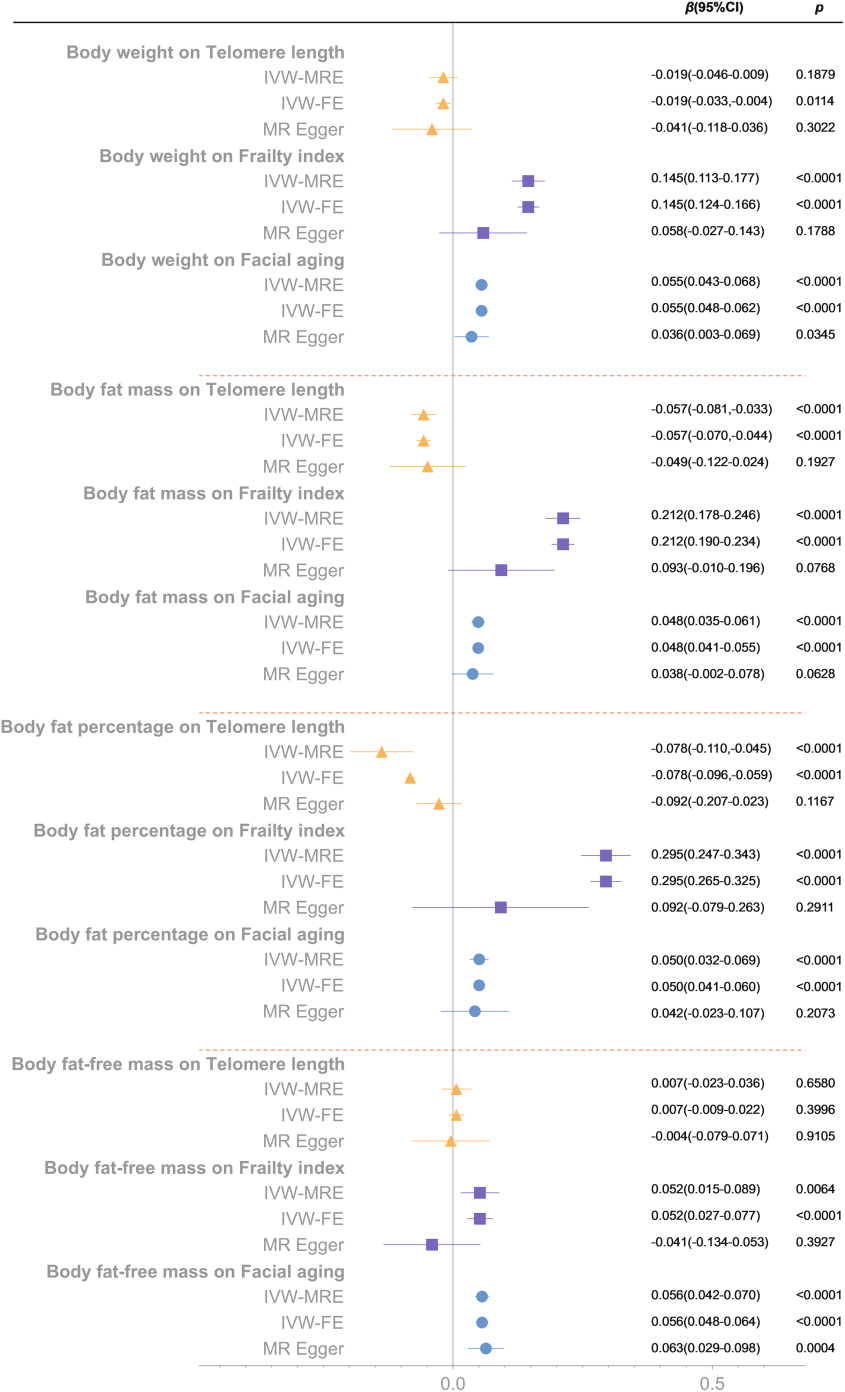
Mendelian randomization analysis of the effect of body weight, body fat mass, body fat percentage and body fat‐free mass on telomere length, frailty index and facial aging. IVW‐MRE, inverse variance weighted (multiplicative random effects); IVW‐FE, inverse variance weighted (fixed effects).

The analyses results showed that both BFM and BFP had significant causal links on aging proxy indicators (BFM on TL: *β* = −0.057, 95% CI = −0.081 to −0.033, *p* < 0.0001; BFM on FI: *β* = 0.212, 95% CI = 0.178–0.246, *p* < 0.0001; BFM on FA: *β* = 0.048, 95% CI = 0.035–0.061, *p* < 0.0001; BFP on TL: *β* = −0.078, 95% CI = −0.110 to −0.045, *p* < 0.0001; BFP on FI: *β* = 0.295, 95% CI = 0.247–0.343, *p* < 0.0001; BFP on FA: *β* = 0.050, 95% CI = 0.032–0.069, *p* < 0.0001). There were causal associations of BW on FI and FA (*β* = 0.145, 95% CI = 0.113–0.177, *p* < 0.0001; *β* = 0.055, 95% CI = 0.043–0.068, *p* < 0.0001 respectively), but not on TL. BFFM only significantly associated with FA (*β* = 0.056, 95% CI = 0.042–0.070, *p* < 0.0001). Although the IVW method showed a causal link between BFFM and FI, the MR‐Egger method showed an opposite direction, suggesting that the causality was invalid (Bowden et al., 2015). (Figure 3).

### 3.6 Obesity indices and aging proxy indicators

The results suggested that waist circumference and waist‐to‐hip ratio had significant positive causal effects on FI (*β* = 0.134, 95% CI = 0.064–0.204, *p* = 0.002; *β* = 0.109, 95% CI = 0.040–0.177, *p* = 0.0019), while hip circumference, waist circumference and waist‐to‐hip ratio had significant positive causal effects on FA (*β* = 0.051, 95% CI = 0.027–0.074, *p* < 0.0001; *β* = 0.055, 95% CI = 0.028–0.082, *p* = 0.0001; *β* = 0.059, 95% CI = 0.025–0.094, *p* = 0.008 respectively). After adjusted for BMI, only waist‐to‐hip ratio was significantly associated with FI, and waist circumference was significantly associated with FA (*β* = 0.062, 95% CI = 0.014–0.111, *p* = 0.0122; *β* = 0.036, 95% CI = 0.011–0.061, *p* = 0.0048 respectively) (Figure S7).

### 3.7 Confounding analysis

**FIGURE 4:**
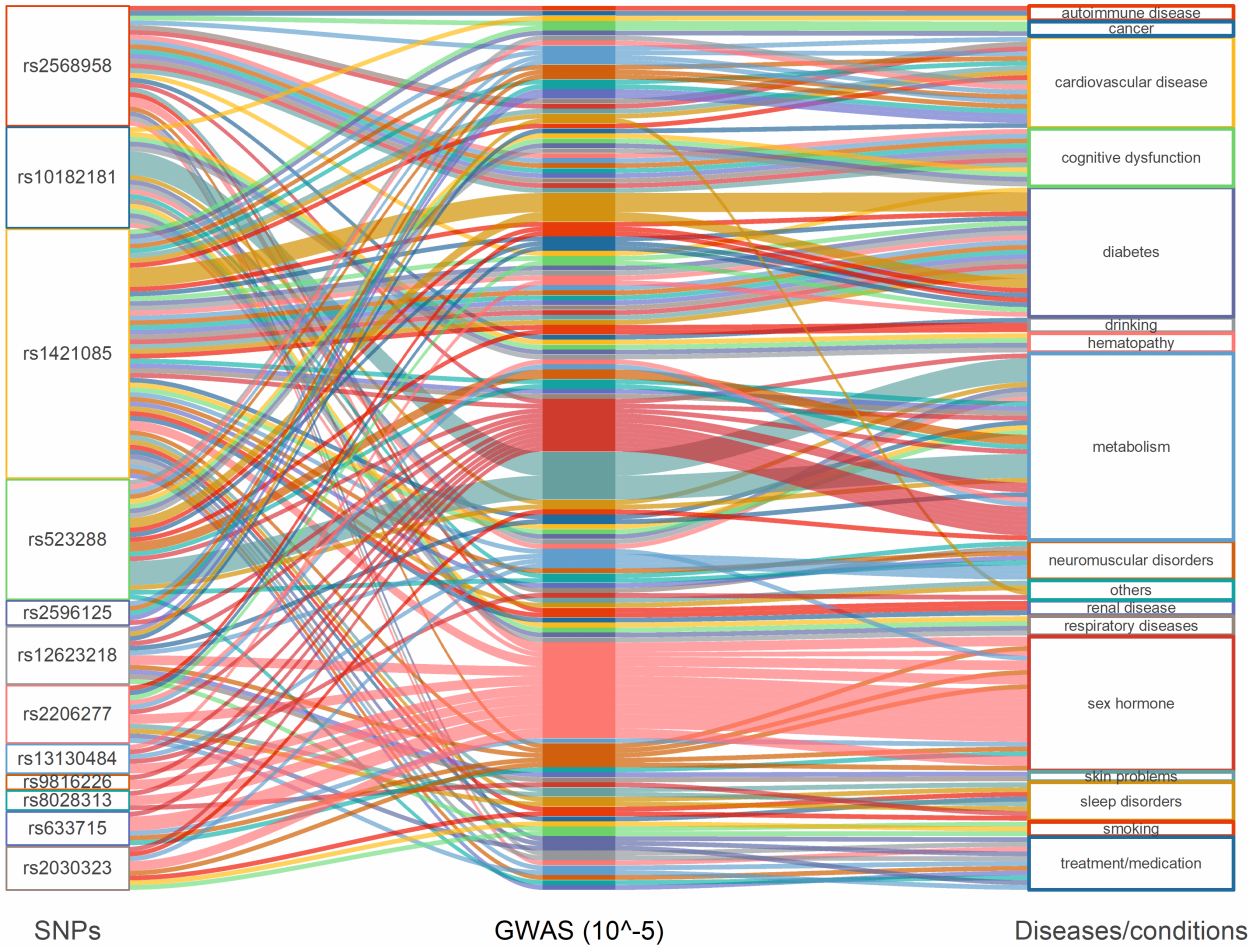
The result of confounding analysis. SNP, single nucleotide polymorphism; GWAS, genome‐wide association studies.

After summarize and analyze the related information about overweight associated SNPs, GWASes and Diseases through Phenoscanner, we found some potential confounding factors, mainly including substance/energy metabolism, sex hormones, cardiovascular diseases, diabetes, cognitive dysfunction, smoking, drinking, renal diseases, respiratory diseases, neuromuscular disorders, autoimmune diseases, and cancer, etc. (Figure 4).

### 3.8 Multivariate analysis

**FIGURE 5:**
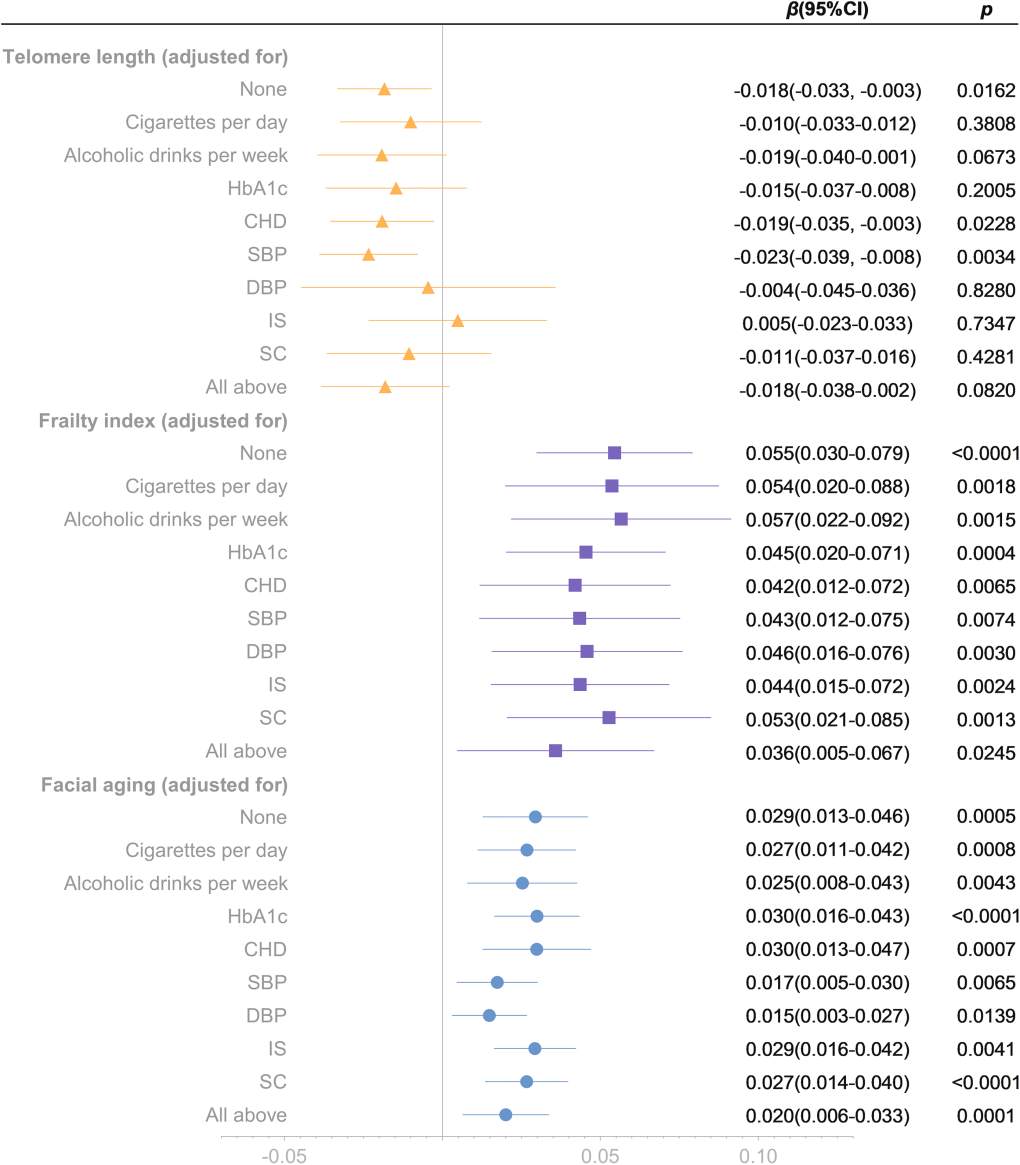
Multivariate analysis of the effects of overweigh on telomere length, frailty index and facial aging. CHD, coronary heart disease; SBP, systolic blood pressure; DBP, diastolic blood pressure; IS, ischemic stroke; SC, serum creatinine.

To further estimate the associations between overweight and aging proxy indicators, we performed multivariate MR Analyses (Figure 5). No single factor could adjust the causal effect of overweight on FI, and overweight on FA (all *p* < 0.025). After adjusted for all factors at once, the causal association of overweight and FA remained significant (*p* = 0.0001), while the association between overweight and FI was suggestive (*p* = 0.0245).

### 3.9 Mediation analysis

**FIGURE 6:**
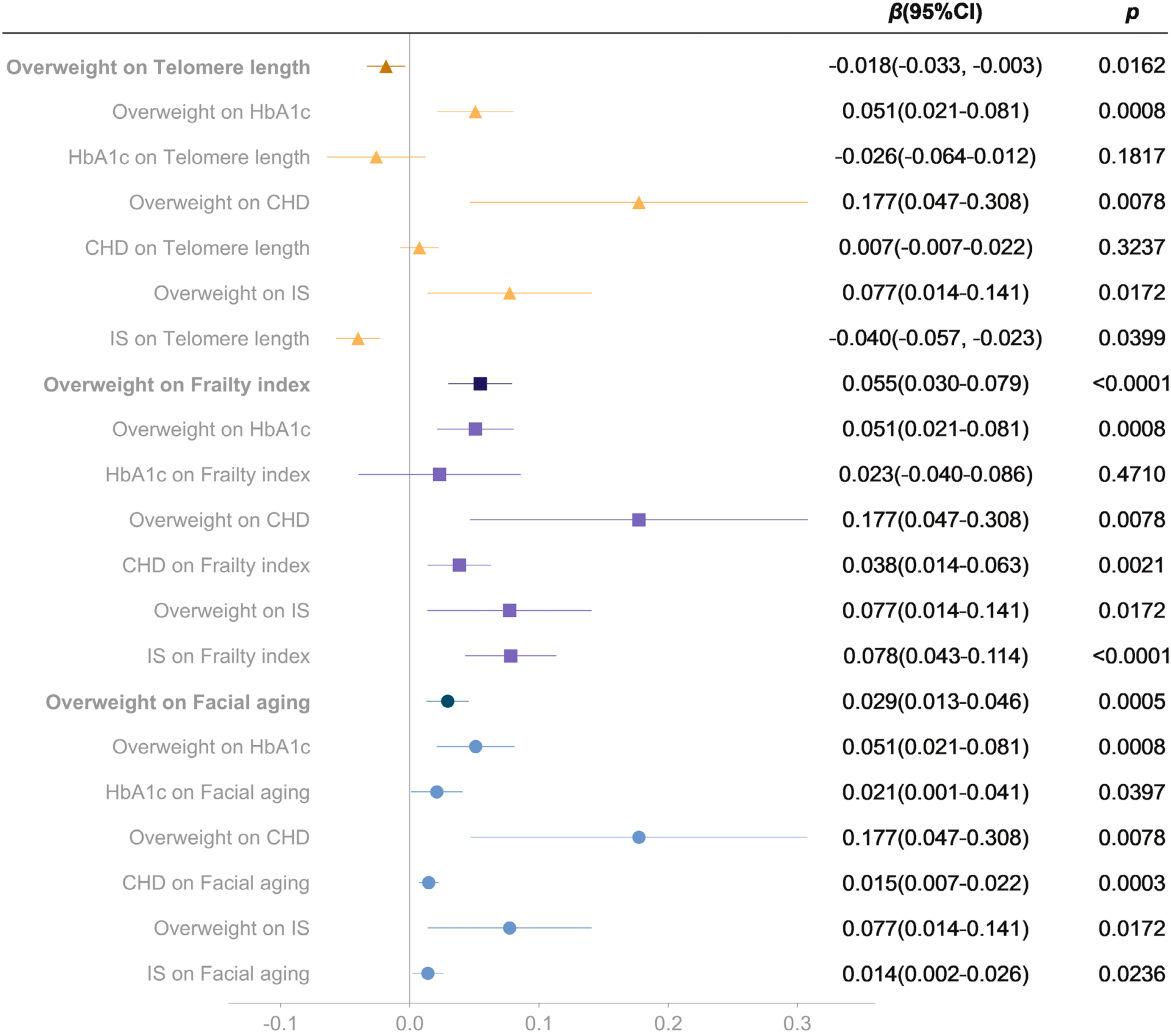
Mediation analysis of the effect of overweight on telomere length, frailty index and facial aging. CHD, coronary heart disease; IS, ischemic stroke.

Based on the results of confounding analysis and multivariate analysis, we analyzed the mediating effects of HbA1c, CHD, and IS on the causality between overweight and aging proxy indicators (Figure 6). The results suggested that the causal effect of overweight on FI might partly mediated by CHD and IS, while the causal link of overweight on FA might partly mediated by HbA1c, CHD and IS. Specifically, CHD and IS mediated approximately 12% and 11% of effects between overweight and FI, respectively. And HbA1c, CHD, and IS mediated approximately 4%, 9% and 4% of effects between overweight and FA, respectively.

## 4 DISCUSSION

In this study, based on the large‐sample GWASes data, we mainly evaluated the associations between overweight and aging proxy indicators using MR analysis. We performed several MR analysis methods including two‐sample analysis, multivariate analysis and mediation analysis, which showed that (1) overweight is probably associated with a higher risk of decreased TL, increased FI, increased FA and decreased longevity expectancy (lower likelihoods to reach or exceed 90th survival percentile and 99th survival percentile); (2) overweight seems to be causally associated with lower parental life expectancy, but the reverse causality was not valid; (3) there is significant causality between HOMA‐IR and overweight, but not with aging proxy indicators; (4) the findings tend to favor causal links between BFM/BFP on aging proxy indicators, but not BFFM; (5) regardless of overweight or not, waist‐to‐hip ratio is a risk factor for increased FI, and waist circumference is a risk factor for increased FA; (6) CHD and IS might mediate the association of overweight on both FI and FA.

Aging is a complex biological process, mainly manifested as TL decrease at the molecular level (Chakravarti et al., 2021). Clinically manifest as frailty, chronic diseases, or abnormal substance/energy metabolism (Rockwood & Howlett, 2018; Stout et al., 2017). In addition, skin aging is an important extrinsic manifestation of aging (Purba et al., 2001; Zou et al., 2021). The definition of aging is based on biological age, and many factors can accelerate aging so that biological age exceeds chronological age (Bhanot et al., 2005). This process is necessarily accompanied by the occurrence of aging related diseases and events, such as increased incidence of chronic diseases or cancer, decreased quality of life, and increased risk of death (Cai et al., 2022). Assessing accelerated aging by measuring the change of biological age proxy indicators including TL, FI, and FA is of great importance for identifying factors contribute to accelerated aging and for intervention. After identifying risk factors, it could help to delay or improve the onset of aging related diseases and events by adjusting medical resource allocation and public health strategies.

Overweight is associated with a variety of acute/chronic diseases, such as hypertension, diabetes, stroke and heart disease (Apovian et al., 2022). It is worth noting that some studies reported that metabolic disorders related to overweight are similar to normal aging, which indicates that overweight may accelerate aging (Robinson et al., 2020). However, accelerated aging is mediated by genes, which is difficult to be identified, and is difficult to intervene. MR analysis is a method to infer causality based on genetic variation, which is suitable for exploring accelerated aging.

In the present study, we collected large‐sample GWASes data like TL, FI and FA from UK Biobank. Then we conducted MR analyses and found that overweight was significantly associated with TL decrease, FI increase and FA increase. TL decrease, FI increase and FA increase could be regarded as the signals of accelerated aging, for which the findings suggested that genetically predicted overweight might be causally related to accelerated aging (Cao et al., 2022; Chakravarti et al., 2021; Stewart & Sharples, 2022). Moreover, the findings suggest that overweight was a risk factor for longevity expectancy, as indicated by the findings that overweight was causally associated with the decreased likelihoods of reaching/exceeding 90th survival percentile and 99th survival percentile, which was consistent with the previous clinical observations (Chen et al., 2019; Hensrud & Klein, 2006). In addition, we explored the associations of BW and BW related parameters (BFM, BFP and BFFM) on aging proxy indicators. Compared with BW, the increase of BFM and BFP was significantly associated with accelerated aging, while the increase of BFFM was not significantly associated with accelerated aging. Taken together, overweight seems to lead to accelerated aging. This might prompt that reducing weight is of great significance for ameliorating the progression of accelerated aging. By extension, public health strategies such as weight control, increasing funding expenditure for overweight and treatment research, increasing the allocation of health resources related to overweight treatment, and developing more rational dietary guidelines are advocated.

By summarizing and analyzing the overweight associated SNPs, GWASes and Diseases, we got some potential confounders and included them in the multivariate analysis. Since confounding and mediating effects share similarities in causal inference, these findings reinforce our understanding of the mechanisms underlying the occurrence of causality and mediation (Carter et al., 2021). The progression of aging is closely related to alterations in substance/energy metabolism, cardiovascular diseases, diabetes, cognitive dysfunction, renal diseases, respiratory diseases, autoimmune diseases, cancer, smoking or alcohol consumption, and so on (López‐Otín et al., 2013). Moreover, the multivariate analysis results showed that the causal effect of overweight on FI and FA was considerably robust, and remained after adjusted for smoking, drinking, HbA1c, CHD, SBP/DBP, IS and SC. In the mediation study, we explored the mediating effect of several FI deficit phenotypes (HbA1c, CHD and IS) on the causality between overweight and aging proxy indicators. The results suggested that CHD and IS might be potential mediators of the causal link of overweight on both FI and FA. Since the insulin resistance plays an important role in the development of overweight (Friesen & Cowan, 2019), we then evaluated the associations of HOMA‐IR with overweight and aging proxy indicators. The results showed that HOMA‐IR had a causal effect on overweight but not on any aging proxy indicator, which could be explained as overweight played a fully mediating role in the association between HOMA‐IR and accelerated aging (Relton & Davey Smith, 2012). Therefore, we need to recognize the importance of weight control as well as improving and treating insulin resistance, so as to cope with the increasing pressure of aging of population and the prevalence of overweight.

Despite the large sample size, our study is prone to several limitations. Although we have used a variety of MR methods to prevent confusion caused by pleiotropy, we cannot completely rule out residual bias, which is the established limitation of MR studies. And MR studies often explore the lifelong impact of risk factors on outcomes, and it is difficult to reveal the causal effects of different stages of disease development. At present, there is no GWAS data related to low BW and no gender stratified GWAS data for overweight, so the association between low BW and aging proxy indicators, and the gender difference in overweight as well as accelerated aging cannot be addressed in this study. Additionally, we need to realize that MR Analysis is definitely less suggestive of causality than RCT, and more high‐quality RCTs evidence is still needed to supplement and support.

## 5 CONCLUSIONS

The present study identified overweight as a risk factor for accelerated aging (TL decreased, FI increased, FA increased) and decreased longevity expectancy. Accordingly, the potential significance of weight control and treatment of overweight in combating accelerated aging need to be emphasized. The findings additionally suggest the necessity of improving and treating insulin resistance against overweight and accelerated aging.

## AUTHOR CONTRIBUTIONS

*Conceptualization*: Xiaolei Jin; *Investigation*: Zong Chen, Zhiyou Chen; *Writing, review, and editing*: all authors.

## CONFLICT OF INTEREST STATEMENT

The authors have no conflicts of interest to declare.

## Supporting information


**Figure. S1.** The leave‐one‐out tests of overweight on aging proxy indicators (telomere length, frailty index and facial aging)

**Figure. S2.** SNPs' effect size of overweight on telomere length

**Figure. S3.** Mendelian randomization analysis of the effect of aging proxy indicators (telomere length, frailty index and facial aging) on overweight

**Figure. S4.** The leave‐one‐out tests of overweight on longevity (90th survival percentile and 99th survival percentile)

**Figure. S5.** Bidirectional Mendelian randomization analysis of the effect between overweight and parental lifespans

**Figure. S6.** Mendelian randomization analysis of the effect of HOMA‐IR on overweight and aging proxy indicators (telomere length, frailty index and facial aging)

**Figure. S7.** Mendelian randomization analysis of the effect of obesity indices on aging proxy indicators (telomere length, frailty index and facial aging)

Table. S1.

## Data Availability

Full GWAS summary statistics for the exposure and outcome data used herein can be found at https://www.ebi.ac.uk/gwas and https://gwas.mrcieu.ac.uk/. The data of human genotype–phenotype associations (SNP‐GWAS‐Disease) could be found at http://www.phenoscanner.medschl.cam.ac.uk/.
