# Supplementary figures and images for: Mendelian randomization supports causality between overweight status and accelerated aging
Source: Aging Cell. 2023 Jun 5;22(8):e13899. doi: 10.1111/acel.13899 (PMC10410004; doi:10.1111/acel.13899)

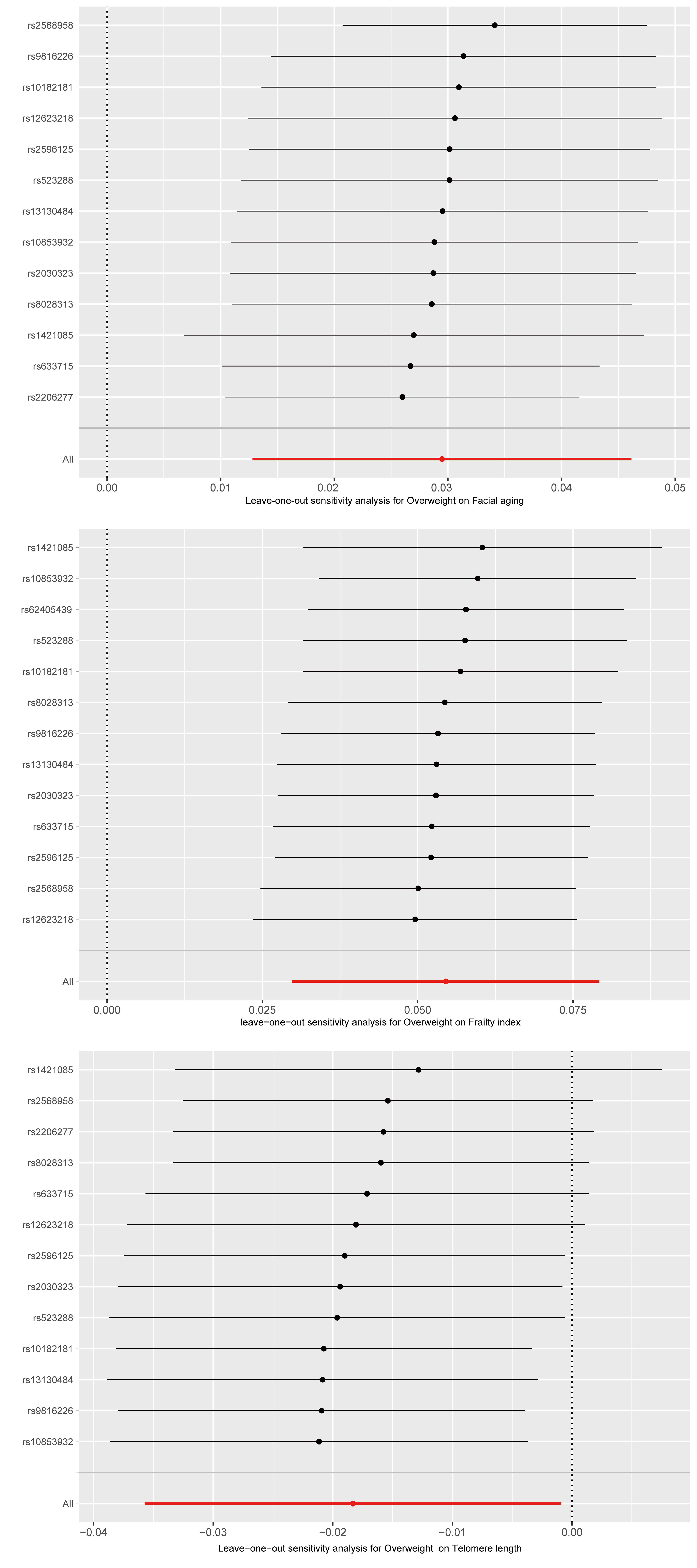

Supplement: Supplementary file 1 — Figure. S1. The leave‐one‐out tests of overweight on aging proxy indicators (telomere length, frailty index and facial aging) [file ACEL-22-e13899-s006.tif]

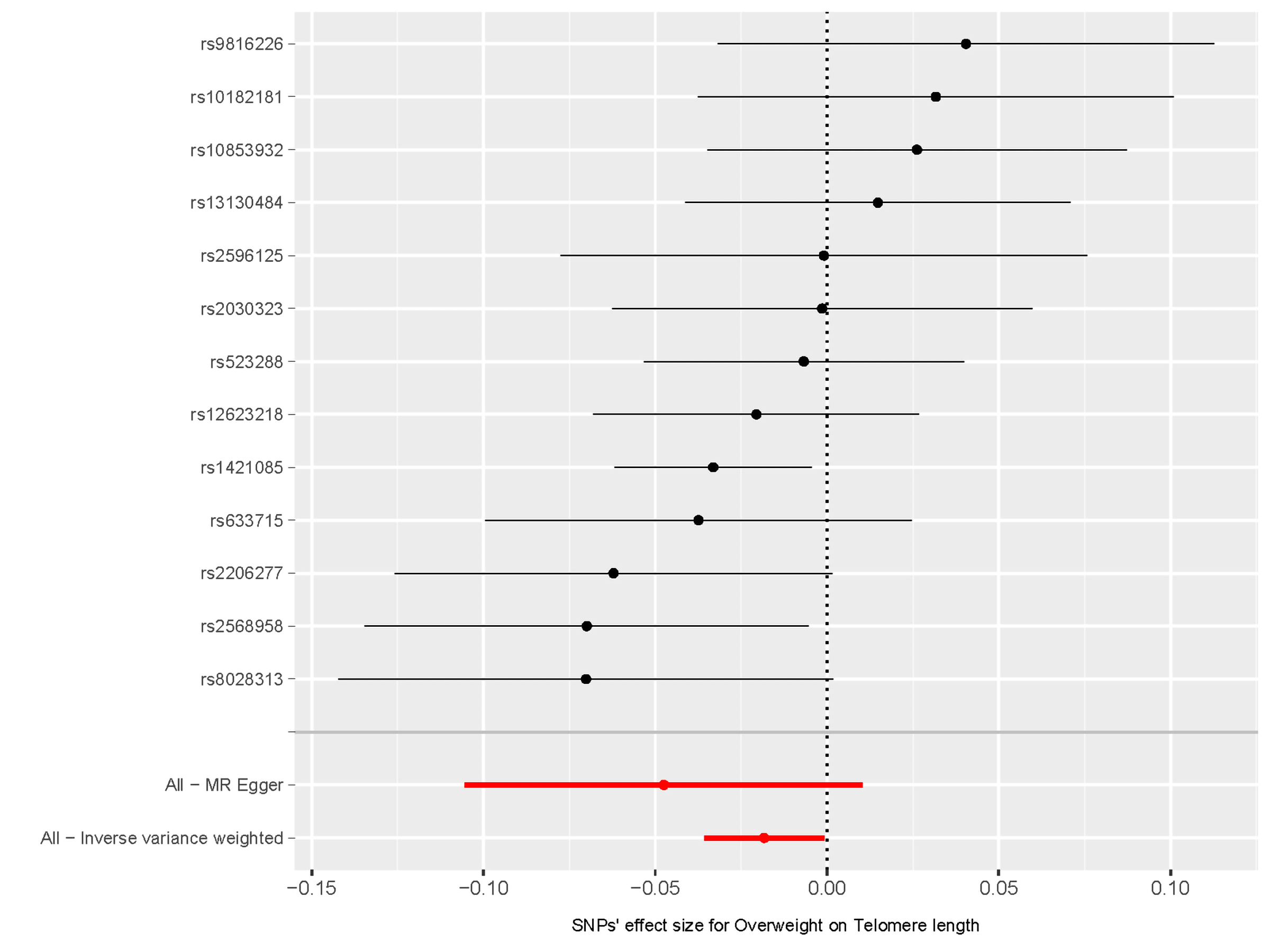

Supplement: Supplementary file 2 — Figure. S2. SNPs' effect size of overweight on telomere length [file ACEL-22-e13899-s005.tif]

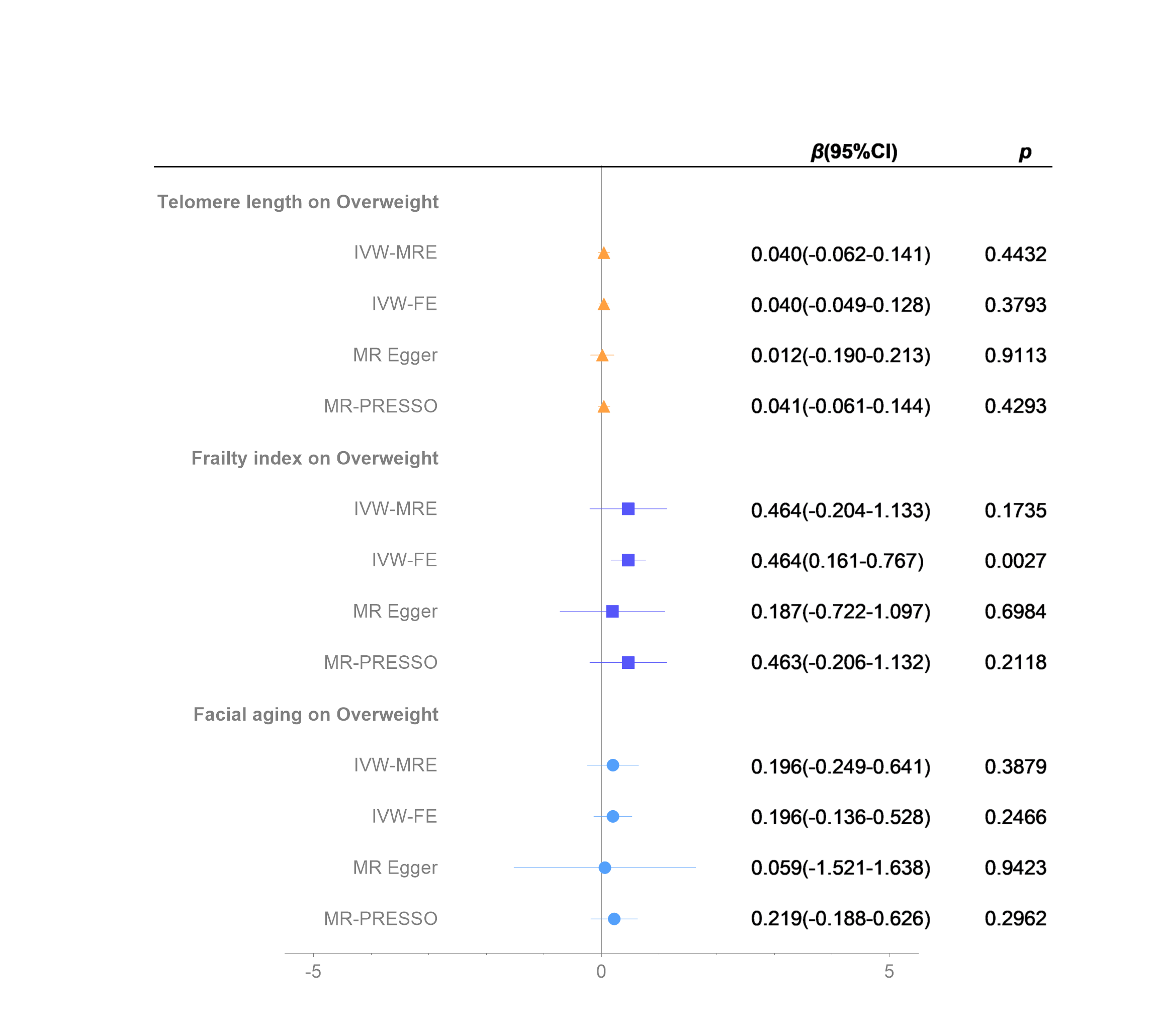

Supplement: Supplementary file 3 — Figure. S3. Mendelian randomization analysis of the effect of aging proxy indicators (telomere length, frailty index and facial aging) on overweight [file ACEL-22-e13899-s001.tif]

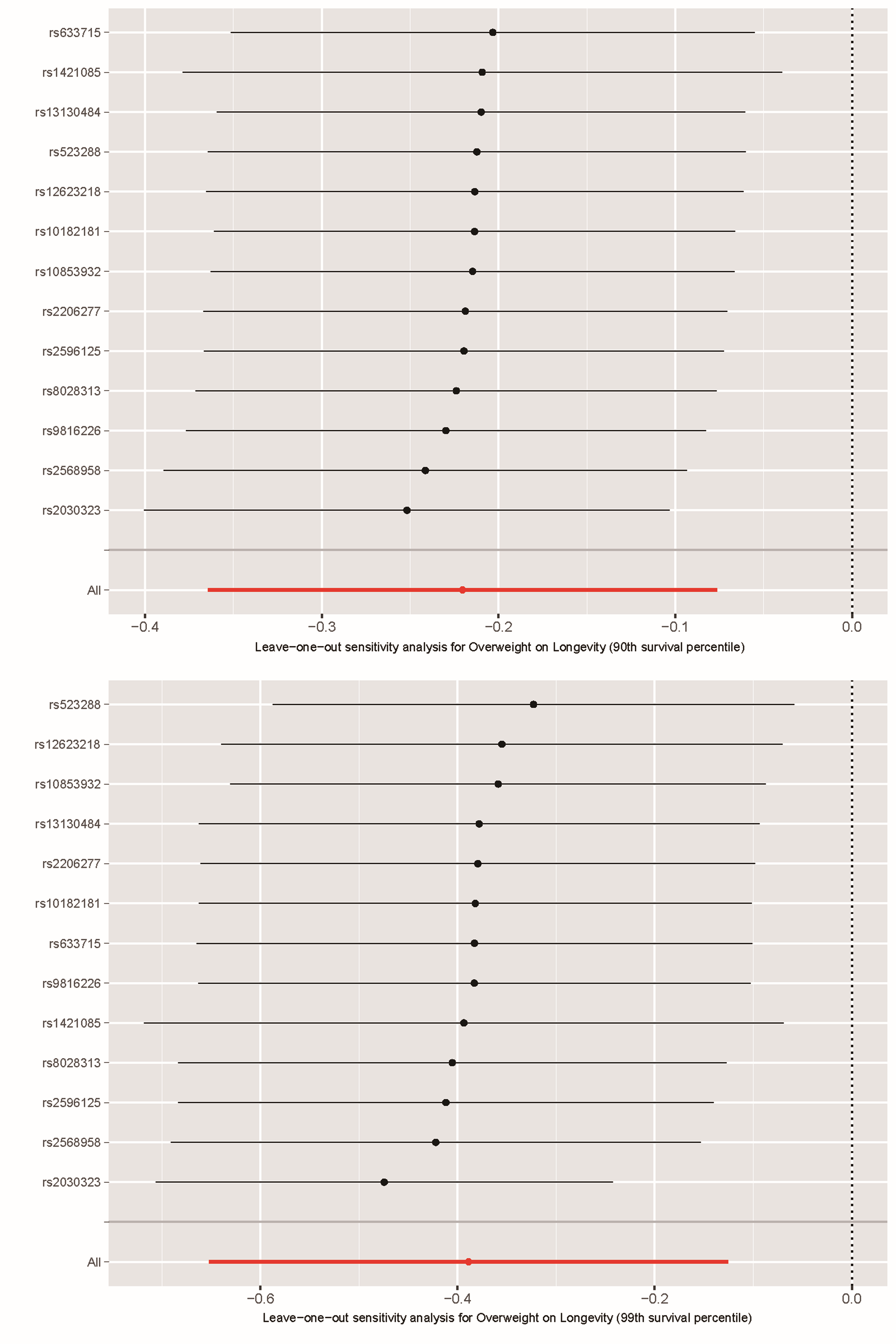

Supplement: Supplementary file 4 — Figure. S4. The leave‐one‐out tests of overweight on longevity (90th survival percentile and 99th survival percentile) [file ACEL-22-e13899-s007.tif]

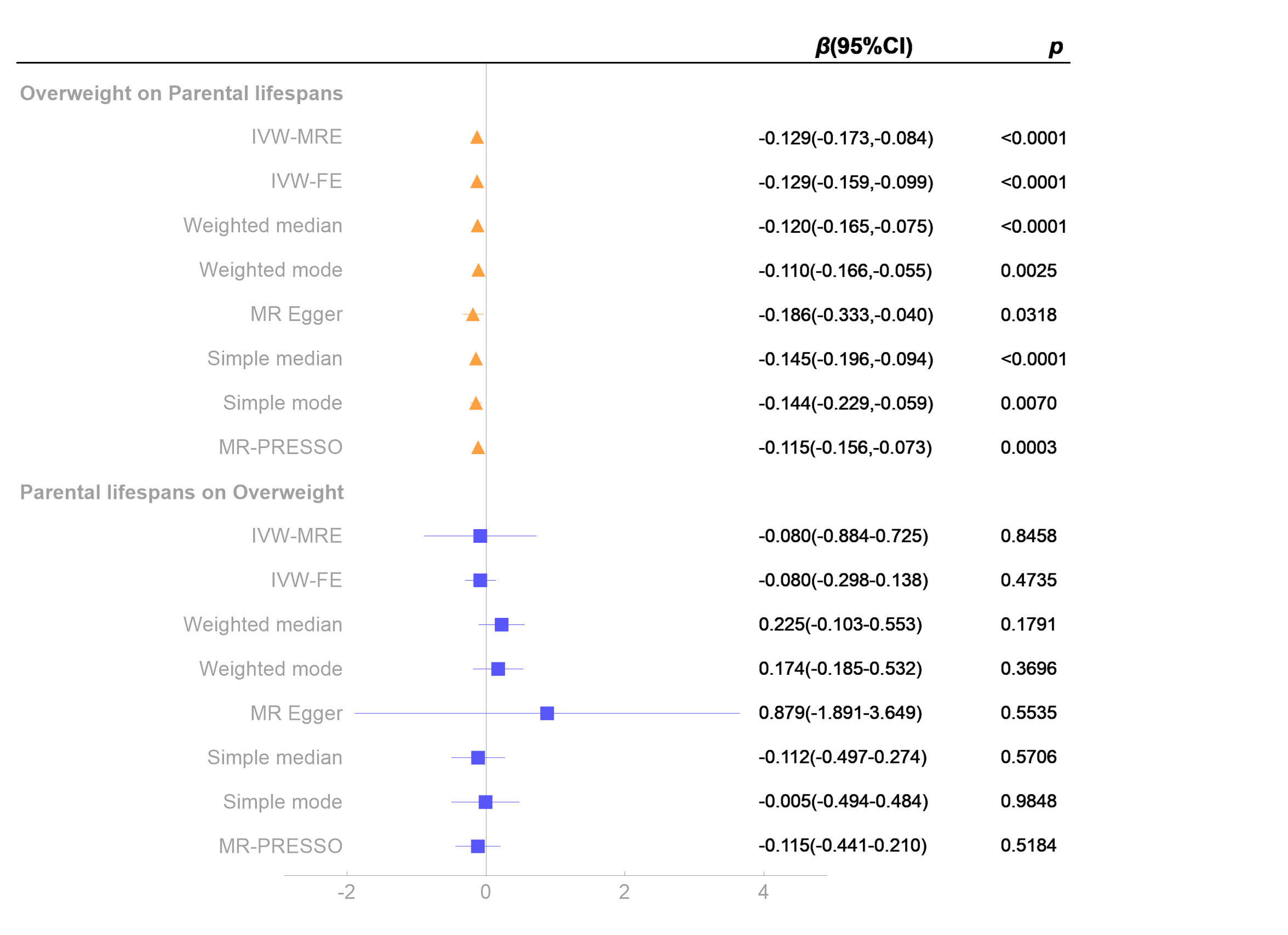

Supplement: Supplementary file 5 — Figure. S5. Bidirectional Mendelian randomization analysis of the effect between overweight and parental lifespans [file ACEL-22-e13899-s002.tif]

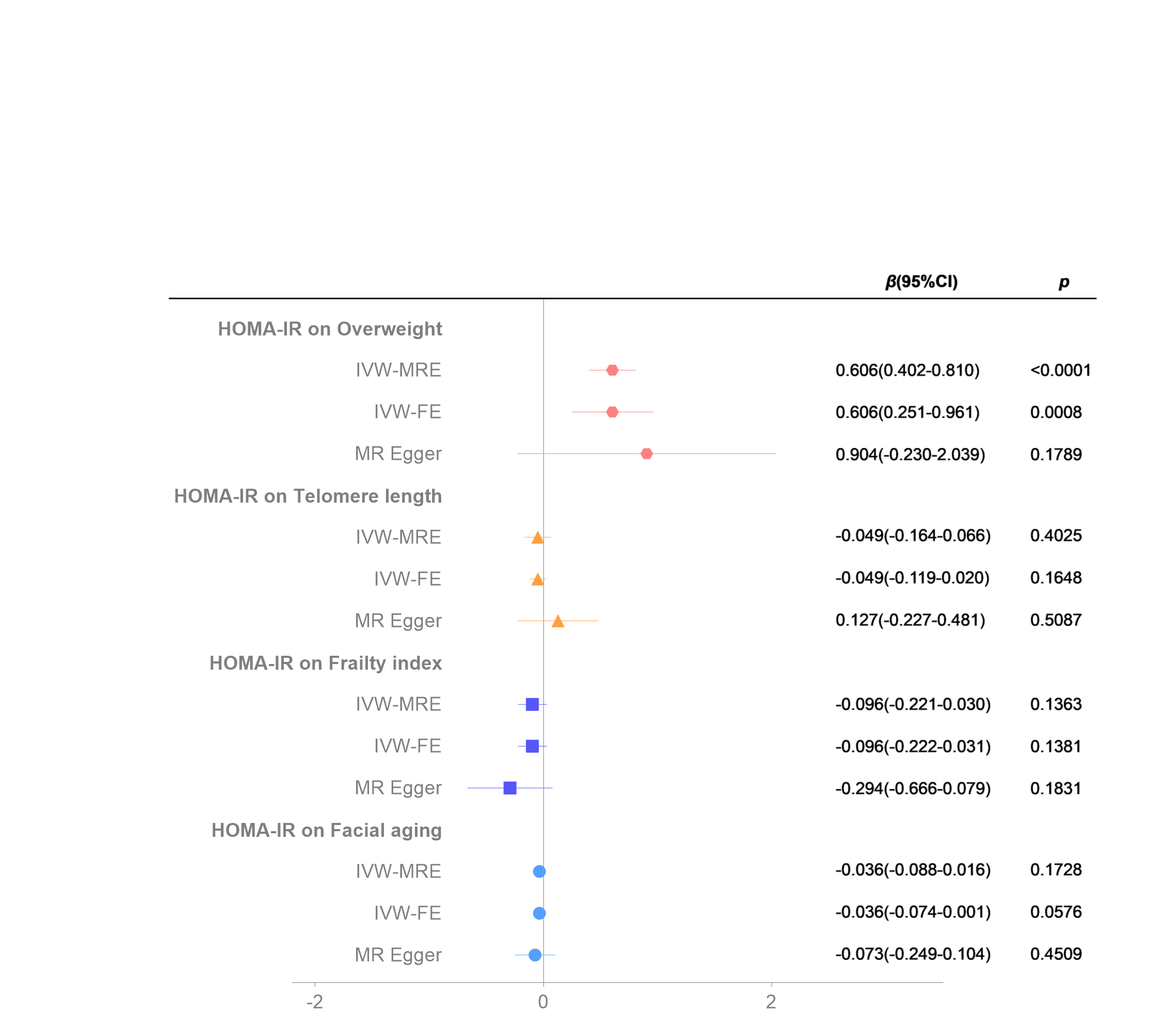

Supplement: Supplementary file 6 — Figure. S6. Mendelian randomization analysis of the effect of HOMA‐IR on overweight and aging proxy indicators (telomere length, frailty index and facial aging) [file ACEL-22-e13899-s004.tif]

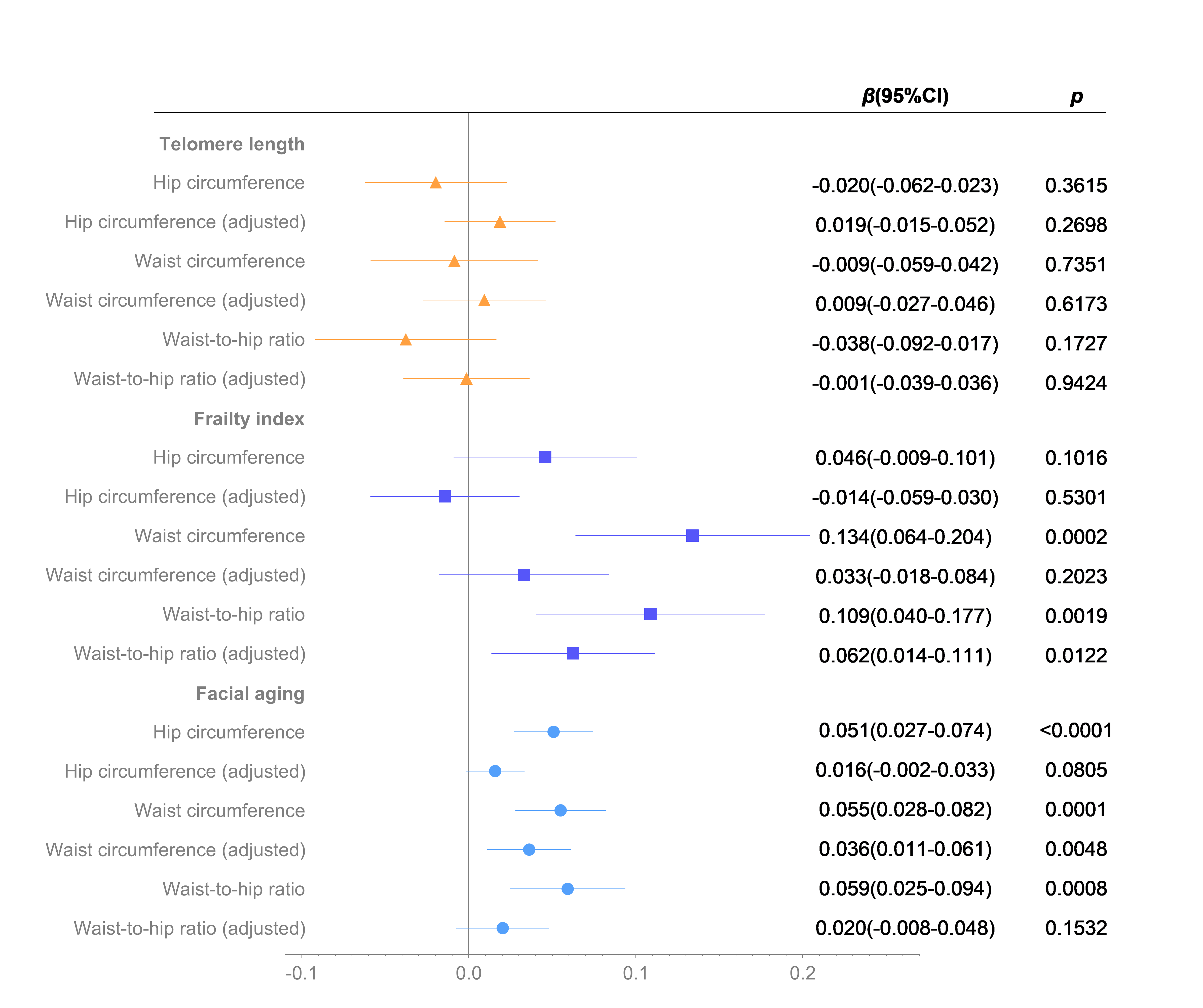

Supplement: Supplementary file 7 — Figure. S7. Mendelian randomization analysis of the effect of obesity indices on aging proxy indicators (telomere length, frailty index and facial aging) [file ACEL-22-e13899-s008.tif]
