# Supplementary material for: Mendelian randomization supports causality between overweight status and accelerated aging
Source: Aging Cell. 2023 Jun 5;22(8):e13899. doi: 10.1111/acel.13899 (PMC10410004; doi:10.1111/acel.13899)
Supplement: Supplementary file 8 — Table. S1. [file ACEL-22-e13899-s003.docx]

| **Type of deficit** | **Item** | **Trait (diseases and conditions)** |
| --- | --- | --- |
| *Sensory* | 1 | Glaucoma |
|  | 2 | Cataracts |
|  | 3 | Hearing difficulty |
| *Cranial* | 4 | Migraine |
|  | 5 | Dental problems |
| *Mental wellbeing* | 6 | Self-rated health |
|  | 7 | Fatigue: frequency of tiredness / lethargy in last two weeks |
|  | 8 | Sleep: experience of sleeplessness/insomnia |
|  | 9 | Depressed feelings: frequency in last two weeks |
|  | 10 | Self-described nervous personality |
|  | 11 | Severe anxiety/ panic attacks |
|  | 12 | Common to feel loneliness |
|  | 13 | Sense of misery (ever/never) |
| *Infirmity* | 14 | Infirmity: long-standing illness or disability |
|  | 15 | Falls in last year |
|  | 16 | Fractures/broken bones in last five years |
| *Cardiometabolic* | 17 | Diabetes |
|  | 18 | Myocardial infarction |
|  | 19 | Angina |
|  | 20 | Stroke |
|  | 21 | High blood pressure |
|  | 22 | Hypothyroidism |
|  | 23 | Deep-vein thrombosis |
|  | 24 | High cholesterol |
| *Respiratory* | 25 | Breathing: wheeze in last year |
|  | 26 | Pneumonia |
|  | 27 | Chronic bronchitis/emphysema |
|  | 28 | Asthma |
| *Musculoskeletal* | 29 | Rheumatoid arthritis |
|  | 30 | Osteoarthritis |
|  | 31 | Gout |
|  | 32 | Osteoporosis |
| *Immunological* | 33 | Hayfever, allergic rhinitis or eczema |
|  | 34 | Psoriasis |
| *Cancer* | 35 | Any cancer diagnosis |
|  | 36 | Multiple cancers diagnosed (number reported) |
| *Pain* | 37 | Chest pain |
|  | 38 | Head and/or neck pain |
|  | 39 | Back pain |
|  | 40 | Stomach/abdominal pain |
|  | 41 | Hip pain |
|  | 42 | Knee pain |
|  | 43 | Whole-body pain |
|  | 44 | Facial pain |
|  | 45 | Sciatica |
| *Gastrointestinal* | 46 | Gastric reflux |
|  | 47 | Hiatus hernia |
|  | 48 | Gall stones |
|  | 49 | Diverticulitis |

Supplementary Material Table 1. Deficit items from the baseline UK Biobank assessment used to compose the Frailty Index.
